# Exercise intensity governs tumor control in mice with breast cancer

**DOI:** 10.3389/fimmu.2024.1339232

**Published:** 2024-03-01

**Authors:** Igor L. Gomes-Santos, Ashwin S. Kumar, Franziska Hausmann, Max N. Meyer, Sarah Z. Shiferaw, Zohreh Amoozgar, Rakesh K. Jain, Dai Fukumura

**Affiliations:** ^1^ Edwin L. Steele Laboratories, Department of Radiation Oncology, Massachusetts General Hospital and Harvard Medical School, Boston, MA, United States; ^2^ Harvard-Massachusetts Institute of Technology (MIT) Division of Health Sciences and Technology, Massachusetts Institute of Technology, Cambridge, MA, United States

**Keywords:** exercise, breast cancer, exercise intensity, tumor microenvironment, immunity, CD8+ T cells

## Abstract

**Introduction:**

Exercise is recommended as an adjunct therapy in cancer, but its effectiveness varies. Our hypothesis is that the benefit depends on the exercise intensity.

**Methods:**

We subjected mice to low intensity (Li), moderate intensity (Mi) or high intensity (Hi) exercise, or untrained control (Co) groups based on their individual maximal running capacity.

**Results:**

We found that exercise intensity played a critical role in tumor control. Only Mi exercise delayed tumor growth and reduced tumor burden, whereas Li or Hi exercise failed to exert similar antitumor effects. While both Li and Mi exercise normalized the tumor vasculature, only Mi exercise increased tumor infiltrated CD8+ T cells, that also displayed enhanced effector function (higher proliferation and expression of CD69, INFγ, GzmB). Moreover, exercise induced an intensity-dependent mobilization of CD8+ T cells into the bloodstream.

**Conclusion:**

These findings shed light on the intricate relationship between exercise intensity and cancer, with implications for personalized and optimal exercise prescriptions for tumor control.

## Introduction

Exercise is a widely recommended adjunct therapy for patients with cancer, offering potential benefits to both physical and psychological well-being. However, the optimal parameters for prescribing exercise as a therapeutic intervention remain a subject of debate (1). Among many variables that may be taken in consideration for aerobic exercise prescription, the intensity of exercise deserves close attention.

Personalizing exercise intensity accounts for the intrinsic variations in individual aerobic capacity and ensures that interventions are tailored to an individual’s relative metabolic demands. This concept, central to exercise physiology (2), holds particular significance in the context of cancer, where a refined approach to exercise prescription is needed.

In preclinical models, we and other researchers have investigated the effects of exercise on the tumor microenvironment (TME) and tumor control (3–8). Notably, our previous report revealed a pivotal role of moderate continuous aerobic exercise in promoting a CD8+ T cell-mediated antitumor response. Central to this phenomenon is the normalization of tumor vasculature, a process that improves vessel maturation and perfusion, facilitates the infiltration of CD8+ T cells into the TME, and supports their antitumor function (9). Indeed, we demonstrated that moderate intensity exercise holds the potential to sensitize otherwise refractory breast tumor models to immune checkpoint blockade (3), a finding with profound clinical implications. Our findings were subsequently corroborated in a similar velocity-controlled exercise regimen where exercise-induced antitumor effects were mediated by CD8+ T cells in an orthotopic pancreatic cancer model (6). By contrast, several preclinical studies of exercise interventions failed to observe a similar increase in CD8+ T cell infiltration or antitumor effects (4, 10), although they showed tumor vessel normalization (3, 4, 7, 8). Such discrepancies entreat explanation.

One commonly proposed argument to reconcile these disparities is the tumor-specific responsiveness to exercise. It was posited that certain tumors may be susceptible to exercise-induced alterations in the TME, while others are not (8, 11). However, a critical factor that deserves scrutiny in those findings is the use of the same running velocity for different mouse strains. Given the substantial differences in exercise capacity among various strains (3), an exercise regimen calibrated to a specific strain may be suboptimal when applied to another strain. In addition, the intrinsic heterogeneity of exercise capacity among individual mice within the same litter (3) further complicates comparisons between studies and their reproducibility.

To address this issue and harmonize the conflicting data surrounding the antitumor effects of exercise in preclinical models, we explored the intricate interplay between exercise intensity and tumor biology and tested the hypothesis that exercise intensity plays a critical role in reprogramming the TME and tumor control.

## Methods and materials

### Experimental procedures

FVB female syngeneic mice were obtained and kept at Cox-7 defined flora animal facility operated by the Edwin L. Steele Laboratories, Department of Radiation Oncology at MGH. Animal studies were approved by the MGH Institutional Animal Care and Use Committee (2009N000135). MCa-M3C breast cancer cell line, generated in our lab (12), were cultured in DMEM medium with 10% FBS supplemented with 4.5 g/L glucose (Corning). 10^5^ MCa-M3C cells were implanted in the 3^rd^ mammary fat pad of 10–12 weeks-old mice. One week after implantation, tumor size was assessed every 2 days by calipers [tumor volume = π/6 x (long axis) x (short axis)^2^]. Exercise interventions started when tumors averaged 100 mm^3^. Mice were then allocated into Control (Co, untrained), Low-intensity (Li), Moderate-intensity (Mi), or High-intensity (Hi) exercise, defined as ~30%, 60%, and 90% of maximal running capacity (3), respectively, on daily sessions of 30 min each for 11 consecutive days, controlling for tumor size and maximal exercise capacity. Illustrations were created with BioRender.com.

### Exercise test and prescription

One week before tumor implantation, mice were subjected to acclimatization on the running treadmill (Panlab/Harvard Apparatus). The acclimatization consisted of running at a low velocity (10 m/min) for 10 minutes on three alternate days. Once mice were familiarized with the treadmill, we performed a maximal exercise test (3). The test started at 6 m/min with 3 m/min increments every 3 minutes, with 0° inclination, until volitional exhaustion. At the exhaustion point, mice were rapidly removed from the running treadmill, and the final velocity (m/min) was recorded. Mice with similar running velocities and tumor size were allocated into different ranges of intensity-controlled exercise interventions: Low-intensity (Li, ~30% of maximum), Moderate-intensity (Mi, ~60% of maximum), or High-intensity (Hi, ~90% of maximum). Exercise training (ExTr) sessions lasted 30 minutes at a given intensity for 11 consecutive days. We established *a priori* 30 seconds of accumulated shock as criteria to stop exercise intervention and remove mice from the treadmill, alternative to volitional exhaustion (maximal exercise test) or during exercise training sessions. Feasibility was assessed by the percentage of completion of the prescribed ExTr program (100% for Li and Mi, 94% for Hi in FVB mice).

### Blood collection and analysis

For peripheral blood mononuclear cells (PBMCs), samples from the submandibular vein were collected into EDTA-coated tubes. On the 10th day of ExTr (acute exercise), blood was collected at the 20^th^ min, when mice were in steady-state exercise, eliciting accurate timing for detection of circulating parameters in response to acute exercise in exercise-trained mice. At that given day, exercise training session lasted 20 min instead of 30 min. In an attempt to minimize the subacute effects of stress, shortened exercise training session and blood withdraw, blood samples were collected again two days later (12^th^ day) at rest, immediately prior sacrifice, and were considered the baseline levels of circulating parameters. Samples were kept on ice until they were centrifuged at 1200 rpm for 10 min at 4°C. The supernatant was collected and snap-frozen in liquid nitrogen for biochemical analysis (cytokines and catecholamines), while the PBMCs cells were slowly frozen in Bambanker (Bulldog Bio) for flow cytometry. Circulating levels of CXCL10, IL15 and IL6 were individually assessed in duplicate (n = 7 to 10 mice per group) for resting levels and pooled (equal plasma amount from n = 6 mice) for screening acute responses to exercise. Cytokines were quantified by cytokine multiplex cytokine assay (V-PLEX Mouse Cytokine 19-Plex Kit, MSD). Epinephrine and norepinephrine were quantified by high-sensitivity 3 catecholamines ELISA kit (BA-E-5600R, Immusmol) in duplicates (n=7-10 per group) and expressed as a percentage of control. For blood lactate, peripheral blood from the tail vein was sampled at the same time points into lactate strips and immediately analyzed using a portable lactimeter (Lactate Plus, Nova Biomedical).

### Tumor vasculature

Briefly, harvested tumors were 4% formalin-fixed overnight and then incubated with 30% sucrose in PBS until sunk. Tumors were then mounted in OCT (Tissue-Tek) and frozen on dry ICE. 20 μm sections were stained with αSMA (Sigma, C6198, 1:100) for pericyte coverage, CD31 (Milipore, MAB1398Z, 1:100) for vessels and DAPI (Vector Labs, 1:1000) for nuclei. Images were acquired using a slide scanner (AxioScan Z1, Zeiss). Unbiased image quantification was conducted using QuPath (13), along with custom software designed in Python. Vessel density was quantified as percentage area positive for CD31+. Pericyte coverage (colocalization of αSMA and CD31), an indicator of vessel maturation, was determined and expressed as a number of mature vessels (CD31+/αSMA+) (cells/mm^2^). Representative images were generated using the software QuPath.

### Flow cytometry

Twenty-four hours after the last exercise session, mice were anesthetized, and tumors were harvested, and samples were processed as previously described (3). Flow cytometry data were obtained using an LSRII flow cytometer (BD Biosciences) and/or Aurora (Cytek) and analyzed with FlowJo software. The double/aggregated cells were gated out using forward scatter area (FSC-A) versus forward scatter width (FSC-W) and side scatter area (SSC-A) versus side scatter width (SSC-W). Different fluorophores conjugated with the following mAb were used: CD45 (30-F11, Biolegend), TCRβ chain (H57-597, Biolegend), CD4 (RM4-5, Biolegend), FOXP3 (150D, Biolegend), CD8a (53-6.7, Biolegend), CD11c (N418, Biolegend), Gr1 (RB6-8C5, Biolegend), MHC-II (M5/114.15.2, BD Biosciences) CD11b (M1/70, Biolegend), B220 (RA3-6B2, Biolegend), F4/80 (BM8, Biolegend), IFNγ (XMG1.2, Biolegend), Granzyme B (GB11, Biolegend), Ki67 (16A8, Biolegend), CD62L (W18021D, Biolegend), CD44 (NIM-R8, Biolegend), CD69 (H1.2F3, Biolegend), CXCR3 (173, Biolegend), IL15Rα (6B4C88, Biolegend), IL6Rα (D7715A7, Biolegend), CD247 (Biolegend, 10F.9G2).

### Statistical analysis

For comparison among groups, we conducted one-way ANOVA. Two-way ANOVA was used for tumor growth analysis and for immune cells in the blood (Rest vs. Exercise). We adopted Tukey *post hoc* test whenever necessary to identify specific group differences. All statistical analyses were performed using GraphPad Prism 10 software. Unless otherwise noted, n=8 to 10 mice per group. Results are presented as Mean ± Standard Error of the Mean. Differences with p ≥ 0.05 were considered statistically significant.

## Results

### Metabolic demand of exercise increases as a function of increments in running velocity

**Figure 1:**
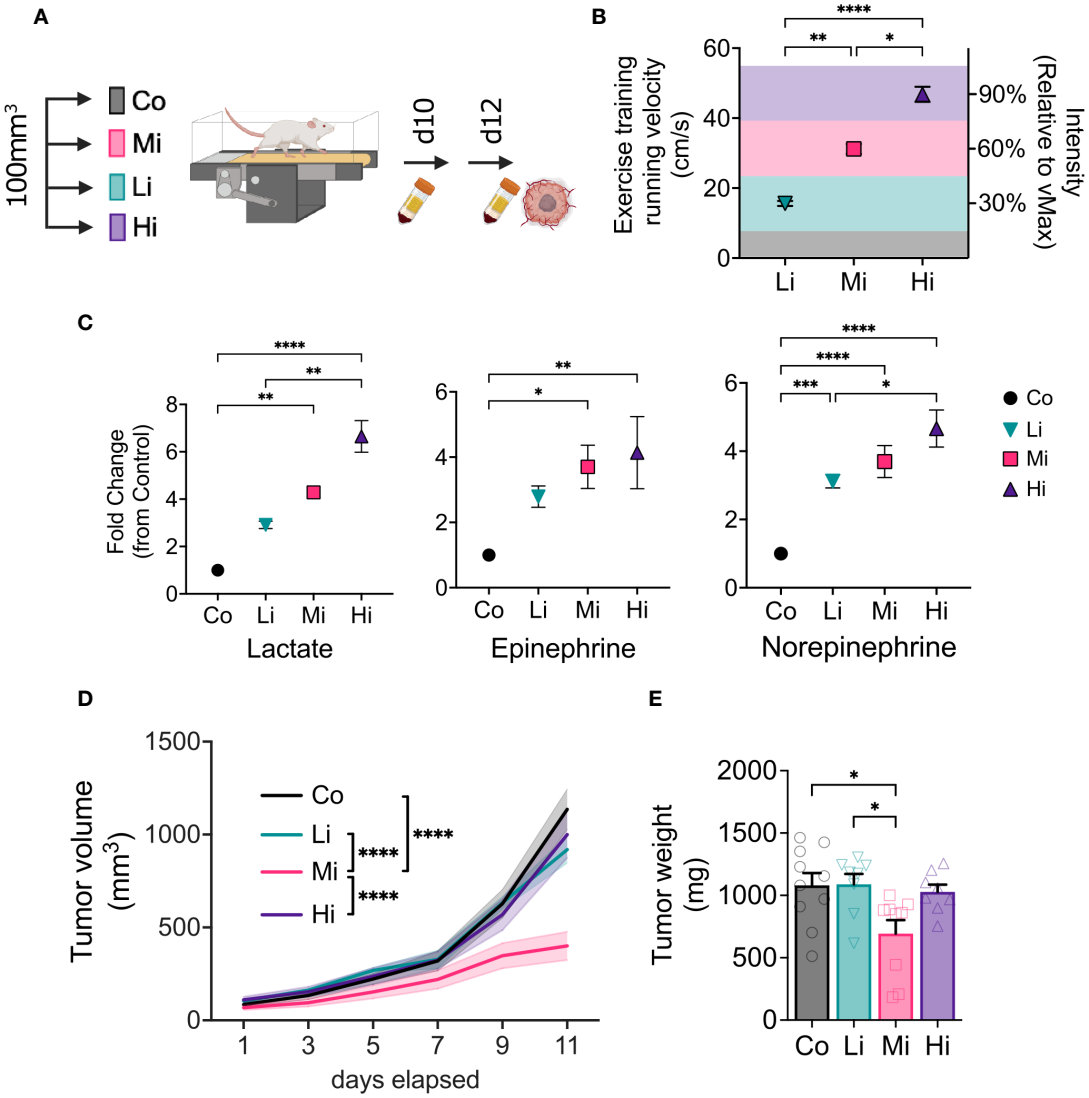
Optimal intensity is required for exercise-induced antitumor effects. **(A)** Experimental design. Syngeneic FVB female mice bearing MCa-M3C orthotopic breast tumors were allocated in 4 different groups when tumors averaged 100 mm^3^: Low-intensity (Li, ~30% of maximum), Moderate-intensity (Mi, ~60% of maximum), or High-intensity (Hi, ~90% of maximum), or Control (Co, untrained) groups. **(B)** Running velocity (in cm/s) and relative intensity (% of maximal exercise capacity, MaEx) for the different exercise training regimens. **(C)** Fold change in circulating levels of Lactate (left panel), Epinephrine (medium panel) and Norepinephrine (right panel; blood collected on day 10 in response to steady-state exercise) in comparison to Control levels (blood collected on day 12 at rest). (n=5-8). **(D)** Tumor growth kinetics and **(E)** final tumor weight in response to different exercise intensities (n=8-10). Error bars show Mean ± SEM. Statistical analysis performed using one-way **(B, C, E)** or two-way **(D)** ANOVAs. ^*^P < 0.05, **P < 0.002, ***P < 0.0002, ****P < 0.0001.

We first determined maximal running capacity of each mouse and used it to prescribe precise exercise interventions, testing different intensities of exercise by adjusting running velocity. At the time of treatment initiation (tumor size ~100 mm^3^), we allocated mice into low-intensity (Li), moderate intensity (Mi) or high-intensity (Hi) (**Figures 1A, B**) of continuous aerobic exercise (at 30%, 60% or 90% of maximal running velocity, respectively), or untrained control (Co). As expected, ten days after the start of exercise training (ExTr), no significant changes of lactate or epinephrine were found among the groups at rest. However, Hi ExTr mice displayed a significant increase in resting levels of norepinephrine in comparison with Co, unexercised mice (**Figures S1D, H**). We then collected blood from mice in steady state running conditions (*i*.*e*., ~20 min progressed from the 10^th^ exercise session). We found that different ExTr velocities triggered distinct and incremental metabolic demand as a function of exercise intensity (assessed by circulating levels of lactate and catecholamines, hallmarks of exercise intensity) (**Figure 1C**). We observed that all doses of exercise were feasible (>95% completion rates, **Figure S1A**), although Hi ExTr mice received significantly more electrical stimuli – potentially causing adverse effects – to sustain the running velocity (**Figures S1B, C**).

### Moderate, but not high or low intensity exercise induces antitumor effect

We measured tumors every other day during treatment. Interestingly, only Mi ExTr group induced tumor control, elicited by delayed tumor growth kinetics (**Figure 1D**) and final tumor weight (**Figure 1E**). Graphic illustrations of tumor growth normalized by initial tumor size (Waterfall plots), time to triple of initial tumor volume, and final tumor volume also confirmed that Li or Hi ExTr do not promote tumor control, which is only achieved by Mi ExTr (**Figures S2A–G**).

### Low and moderate intensity ExTr normalize tumor vasculature

**Figure 2:**
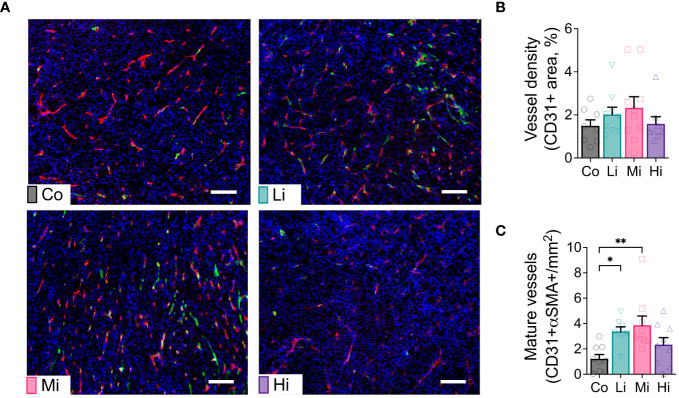
Exercise at Li and Mi normalizes tumor vasculature. **(A)** Representative histological sections of Co (upper-left panel), Li (upper-right panel), Mi (bottom-left panel) and Hi (bottom-right panel) showing vessels (CD31+ staining, red) and pericyte coverage (αSMA staining, green) among tumor cells (DAPI, staining cell nucleus, blue). The white line indicates 100 μm. **(B)** quantification of vessel density and **(C)** mature/normalized vessels (n=8-10). Error bars show Mean ± SEM. Statistical analysis was performed using one-way ANOVA. ^*^P < 0.05, **P < 0.002.

To test the effect of ExTr with different intensities on tumor vasculature, we collected MCa-M3C tumors from FVB mice subjected to different ExTr intensities and determined tumor vascular phenotype by immunostaining. We assessed vessel maturation by measuring the tumor vessels displaying pericyte coverage (double positive for CD31 and αSMA). We found significantly increased CD31-αSMA double positive areas (vessel maturation) in both Li and Mi ExTr. However, this phenotype was not significant in Hi ExTr (**Figures 2A–C**).

### Moderate intensity exercise increases CD8+ T cells displaying enhanced effector phenotype

**Figure 3:**
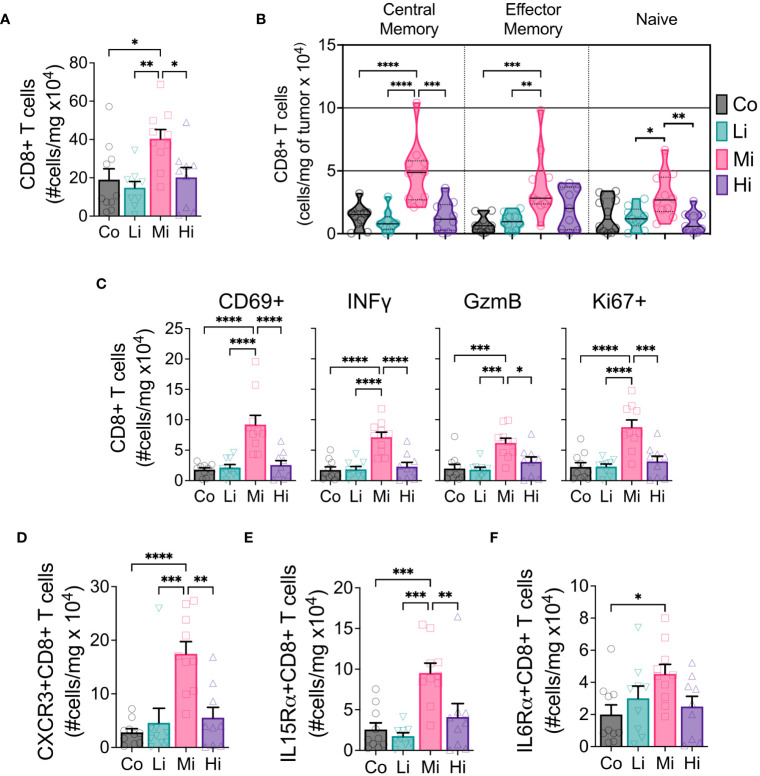
CD8+ T cells displaying enhanced effector phenotype increase with moderate intensity exercise. **(A)** Absolute numbers of CD8+ T cells in tumors. **(B)** Developmental stage of CD8+ T cells (violin plot) indicating Central memory (CD62L+CD44+, left panel), Effector memory (CD62L-CD44+, medium panel) and Naïve (CD62L-CD44-, right panel) status, in absolute numbers. **(C)** Panel displaying the absolute number of CD8+ T cells expressing activation marker CD69, cytotoxic function markers IFNγ and GzmB, and proliferation marker Ki67. Exercise-mediated tumoral enrichment of CD8+ T cells expressing **(D)** CXCR3, **(E)** IL15Rα, and **(F)** IL6Rα, in absolute numbers (n=8-10). Error bars show Mean ± SEM. Statistical analysis was performed using one-way ANOVA. ^*^P < 0.05, **P < 0.002, ***P < 0.0002, ****P < 0.0001.

To examine the effects of different ExTr intensities on the immune TME, we performed flow cytometry of samples from these tumors. We found a marked increase in CD8+ T cells in the TME only in Mi ExTr group (**Figure 3A**, **S3A–C**). Furthermore, only Mi ExTr tumors displayed higher levels of central and effector memory CD8 T+ cells (absolute numbers in **Figure 3B**, relative numbers in **Figure S3D–F**) as well as higher effector function phenotype, characterized by higher expressions of CD69, INFγ, GzmB and proliferation (Ki67+) (**Figure 3C**, **Figure S3G**). We then interrogated whether intensity modulated the profile of CD8+ T cells recruited to tumors. Here again, we observed an enrichment of CXCR3-, IL15Rα- and IL6Rα- receptors in CD8+ T cells within the TME of mice subjected to Mi exercise (**Figures 3D–F**, **Figure S3G**). While both absolute and relative numbers of CD8+ T cells increased with Mi ExTr, the relative number of macrophages among immune cells was significantly reduced in both Mi and Hi ExTr (**Figure 3**, **Figure S4E**), with sustained M1/M2 ratio (**Figure S4I**). Interestingly, we observed an enrichment of DC1 (CD11b-CD11c+CD103+ dendritic cells) in Mi compared to Co and Hi ExTr (**Figure S4M–O**).

Considering the effector function of CD8+ T cells can be profoundly influenced by the expression of exhaustion markers, we tested the expression of PD1, CTLA4, Tim3 and Lag3 in CD8+ T cells and PDL1 in non-immune cells (CD45-) in the TME (**Figure S5**). We found a significant increase of the absolute numbers of CD8+ T cells expressing CTLA4 and Tim3 but not PD1 or Lag3, although Mi ExTr induced a lower fraction of CD8+ T cells expressing PD1. Because PD1 can also be an early activation marker, we analyzed the fraction of CD8+ T cells that were co-expressing PD1 with any of the other exhaustion markers. We found Mi and Hi ExTr (but not Li) significantly reduced CD8+ T cells positive for both PD1 and CTLA4, and Mi reduced the PD1+Tim3+ CD8+ T cells (**Figure S5M**).

### Acute exercise triggers the mobilization of CD8+ T cells expressing multiple recruitment pathways

**Figure 4:**
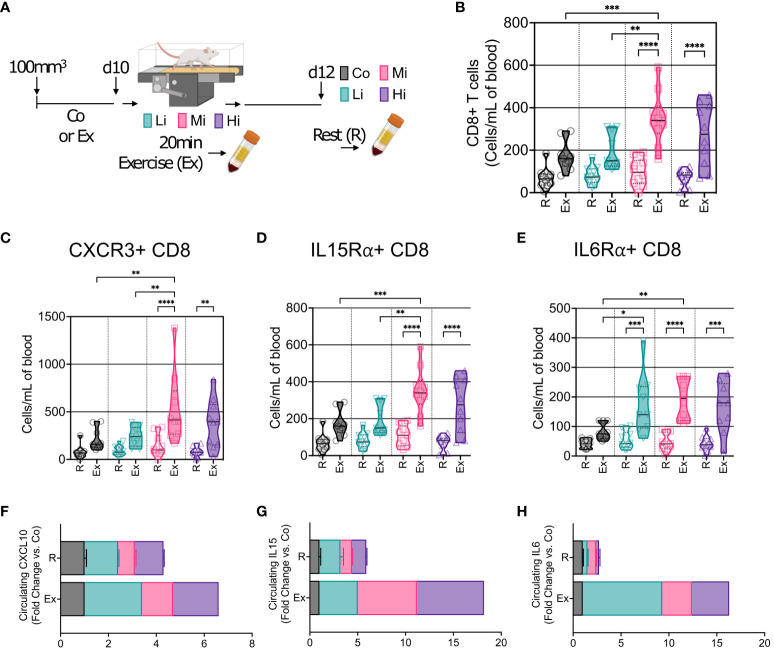
Acute exercise triggers the mobilization of CD8+ T cells expressing multiple recruitment pathways. **(A)** Representation of blood collection approach. After 10 days of exercise training or follow-up, mice in the trained groups were submitted to 20 min of exercise. Peripheral blood was collected immediately after mice were removed from the treadmill (Exercise, Ex). Two days later, blood was collected from all groups prior to sacrifice (Rest, R). **(B)** Number of circulating CD8+ T cells at rest (R) and in response to exercise at different intensities (Ex). Exercise-mediated mobilization of CD8+ T cells expressing **(C)** CXCR3, **(D)** IL15Rα, and **(E)** IL6Rα, in absolute numbers (n=8-10). Screening of circulating levels **(F)** CXCL10 (CXCR3 receptor ligand), **(G)** IL15 (IL15Rα ligand), and **(H)** IL6 (IL6Rα ligand) at rest (R), and in response to exercise (Ex) at different intensities (pooled n = 7 to 10 mice per group, run in duplicate) by cytokine multiplex cytokine assay. Error bars show Mean ± SEM. Statistical analysis was performed using one-way ANOVA. ^*^P < 0.05, **P < 0.002, ***P < 0.0002, ****P < 0.0001.

We then enquired about the mobilization of immune cells into the circulation at different exercise intensity levels. We characterized CD8+ T cells by flow cytometry and found no changes in CD8+ T cells in the blood at rest – several days after ExTr. However, during exercise, we found that Mi and Hi, but not Li exercise, mobilized CD8+ T cells into the bloodstream of mice (**Figures 4A, B**, **Figures S6A, B**). Furthermore, CD8+ T cells expressing CXCR3, IL15Rα and IL6Rα receptors increased after ExTr consistent with the TME immune reprogramming (**Figures 4C–E**, **Figures S6C–H**). Corresponding to the increase in CD8+ T cells, Mi exercise transiently increased circulating levels of ligands to CXCR3 (CXCL10), IL15Rα (IL15) and IL6Rα (IL6) after ExTr, that returned to normal levels (not different from Co) at rest (**Figures 4F–H**).

## Discussion

Exercise is widely recommended as an adjunct therapy to patients with cancer (1, 14, 15), yet the biological effects of exercise on tumor biology remain enigmatic. Our study uncovered the ideal exercise levels that trigger distinct biological responses, challenging previously assumed synchronicity. For instance, functional tumor vessels are essential to promote innate and adaptive immune responses and potentiate immunotherapy for tumor control (16, 17). Our study agrees with previous findings that exercise can normalize the tumor vasculature (3, 4, 7). Moreover, our study confirms that vessel normalization alone is not sufficient for tumor control. Specifically, we found that both Li and Mi ExTr normalized tumor vasculature, but only Mi led to immunostimulatory TME and tumor control.

The exercise-induced mobilization of immune cells in our study recapitulates the behavior observed in human subjects in response to incremental exercise (18). As exercise intensity increases, several regulatory mechanisms come into play to support the demands of exercising muscles and to restore body homeostasis. These mechanisms include higher levels of body temperature (19, 20), sympathetic activation (21, 22), and metabolic rate, all of which can stimulate mobilization of immune cells into the circulation from major lymphoid organs. This aligns our findings with the current understanding that exercise facilitates immune cell mobilization and their redistribution to different organs and tissues (22, 23), including tumors.

Notably, Li ExTr did not yield substantial immune cell recruitment, possibly failing to reach the threshold for sympathetic-system-mediated immune cell mobilization. Circulating levels of norepinephrine at rest increased in Hi. Given that aerobic exercise is well-documented to reduce sympathetic overactivation in chronic diseases (24), this paradoxical finding suggests a potential trigger of chronic stress induced by Hi exercise. This might have been exacerbated by higher electrical stimuli accumulated by Hi ExTr, in association with the higher sympathetic activation induced by Hi ExTr in mice. It remains to be determined whether high-intensity *interval* aerobic exercise can be better tolerated and further enhance Mi ExTr response without triggering sympathetic overactivity.

The boost in CD8+ T cell infiltration in solid tumors, along with the enhanced effector-like phenotype, is intriguing. While the intrinsic effects of exercise on T cell fitness remain a promising yet unexplored area, we recognize that poor perfusion (16), high acidosis (25), and metabolic competition for substrates in highly glycolytic tumors (26, 27) can impair CD8+ T cell effector function. Furthermore, we and others demonstrated that exercise improves tumor perfusion, reduces hypoxia (3, 4, 7), and shifts tumor metabolic signatures towards oxidative phosphorylation (3). Along these lines, low-dose antiangiogenic agents can induce tumor vessel normalization, revive antitumor immunity and boost cytotoxic cell function (17), including those of adoptively transferred CAR-T cells (28).

Our study is consistent with existing knowledge regarding three pathways underlying exercise-induced mobilization and accumulation of cytotoxic cells to the TME: the CXCR3 chemokine (3), IL15Rα (6), and IL6α (29) pathways. More importantly, we show they concur and are dependent on the exercise intensity. Indeed, the profile of tumor-infiltrated lymphocytes is mirrored by circulating lymphocytes at acute exercise but diverges from the profile at rest, which opens possibilities for biomarker exploration in clinical studies.

It is important to note that our intent is not to oversimplify the multifaceted benefits of physical activity on human health. While exercise as a treatment might display dose-dependent effects, we recognize that even small increments of exercise appear to offer a myriad of advantages, including the management of side effects and the promotion of a better quality of life (1), while increasing survival (14, 15). Notably, Li exercise appears to be effective in mitigating cancer-related fatigue (30), a prevalent side effect in oncology. Furthermore, emerging evidence suggests the potential benefits of Hi exercise in cancer survivorship (13). Our findings should thus be viewed as contributing to a standardization of preclinical exercise interventions in mice and encouraging similar awareness in clinical studies. As technological capabilities of monitoring patient’s biopotentials (e.g., physical activity levels, heart rate, body temperature) advance, the integration of exercise intensity into data analysis (31) and exercise prescription is becoming more feasible and clinically relevant.

## Data availability statement

The original contributions presented in the study are included in the article/**Supplementary Material**. Further inquiries can be directed to the corresponding author.

## Ethics statement

The animal study was approved by MGH Institutional Animal Care and Use Committee (#2009N000135). The study was conducted in accordance with the local legislation and institutional requirements.

## Author contributions

IG-S: Conceptualization, Data curation, Formal analysis, Investigation, Methodology, Project administration, Resources, Validation, Visualization, Writing – original draft, Writing – review & editing. AK: Data curation, Formal analysis, Investigation, Methodology, Writing – review & editing. FH: Investigation, Writing – review & editing. MM: Investigation, Writing – review & editing. SS: Investigation, Writing – review & editing. ZA: Conceptualization, Investigation, Supervision, Writing – review & editing, Writing – original draft. RJ: Resources, Supervision, Writing – review & editing. DF: Resources, Supervision, Writing – review & editing.
