# Supplementary material for: Exercise intensity governs tumor control in mice with breast cancer
Source: Front Immunol. 2024 Mar 1;15:1339232. doi: 10.3389/fimmu.2024.1339232 (PMC10940460; doi:10.3389/fimmu.2024.1339232)
Supplement: Supplementary file 1 [file Presentation_1.pdf]

SUPPLEMENT

Exercise intensity governs tumor control in mice with breast cancer

Igor L. Gomes-Santos[1], Ashwin S. Kumar[1,2], Sarah Shiferaw[1], Franziska Hausmann[1,a], Max N. Meyer[1], Zohreh Amoozgar[1,b], Rakesh K. Jain[1*], Dai Fukumura[1*]

[1]Edwin L. Steele Laboratories, Department of Radiation Oncology, Massachusetts General Hospital and Harvard Medical School, Boston MA, USA
[2]Harvard-MIT Division of Health Sciences and Technology, Massachusetts Institute of Technology, Cambridge MA, USA
Current address:
[a]Department of Radiation Oncology and Radiotherapy, Charité University Medicine Berlin, Berlin, Germany
[b]Sanofi, 350 water street, Cambridge MA, USA
*Corresponding authors

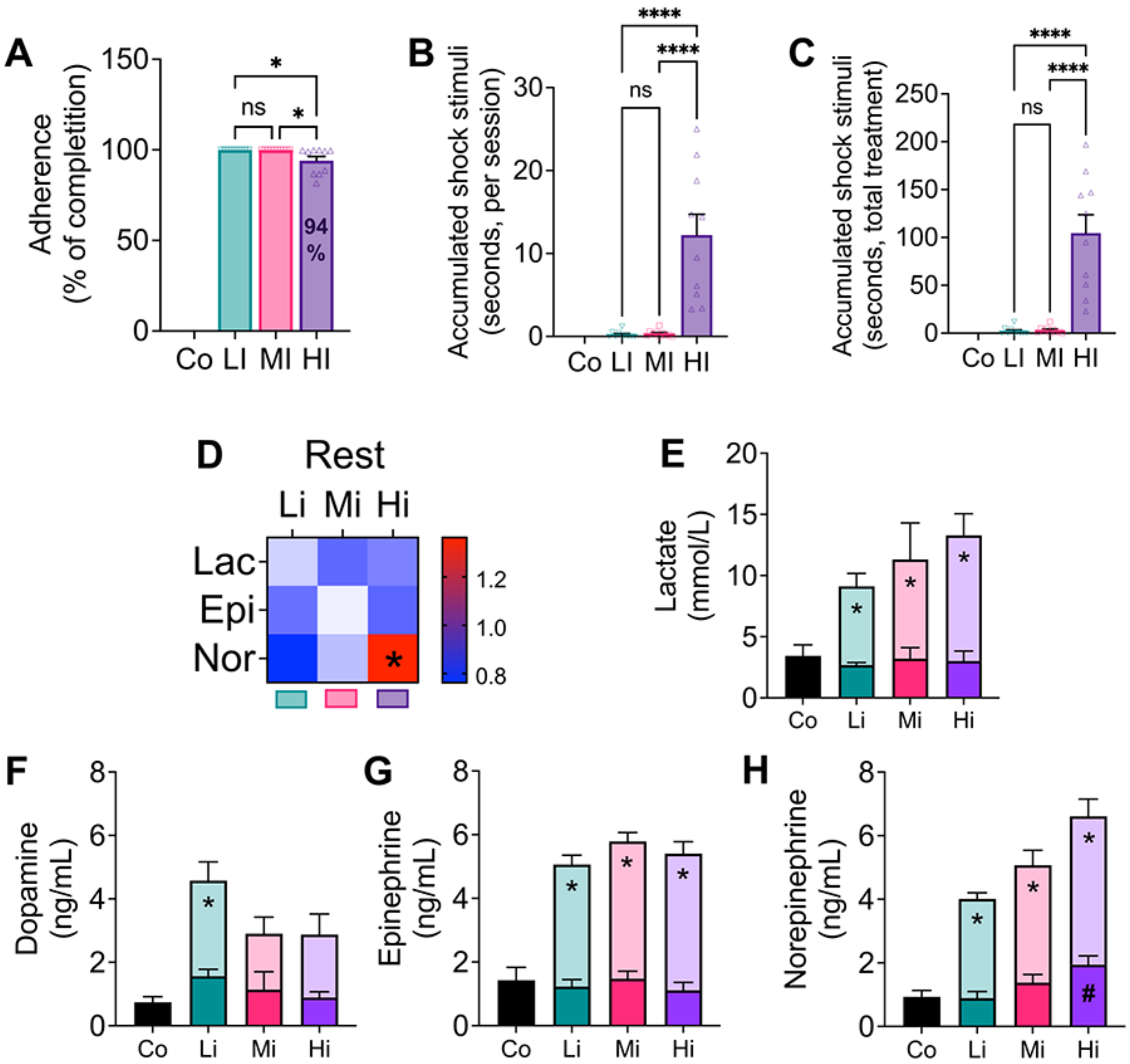

Figure S1. Adherence to exercise training and metabolic demands of different exercise intensities. (**A**) Adherence to exercise, measured by the accumulated competition rate of exercise sessions. (**B**) Average amount of shock stimuli per running session and (**C**) accumulated throughout the study, in seconds (n=9-10). (**D**) Heat map showing circulating levels of lactate (Lac), Epinephrine (Epi) and Norepinephrine (Nor) in comparison to Control group (Co) (**E**). Circulating levels of Lactate (F), Dopamine (G), Epinephrine and (H) Norepinephrine at rest (strong color, bottom bar) and in response to exercise at different intensities (light color, top bar) (n=5-8). For determination of circulating responses to acute exercise, blood was collected on day 10 in response to steady-state exercise, and compared to the resting levels in the Co group, as well as the same exercised mice in each group (blood collected on day 12 at rest). Error bars show Mean ± SEM. Statistical analysis performed using one-way (A-D) or two-way (**E-H**) ANOVAs. On E-H, *P<0.05 and ****P<0.0001. vs. resting levels on the same mice; #P<0.05 vs Co mice at Rest.

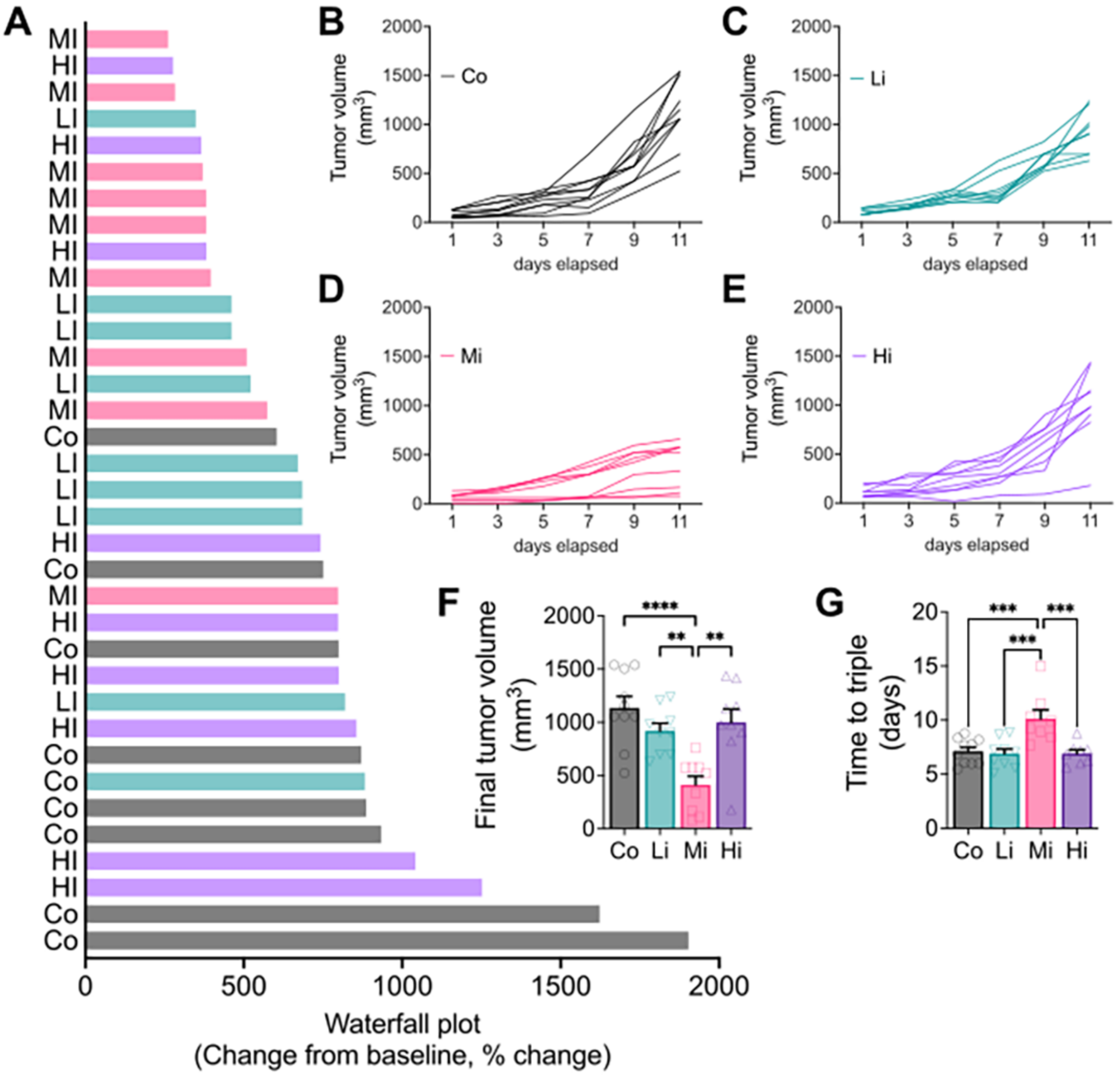

Figure S2. Role of exercise intensity on tumor burden. (**A**) Waterfall plot showing individual mice response to ExTr at different intensities. (**B-E**) Individual tumor growth kinetics in Co (**B**), Li (**C**), Mi (**D**), and Hi (**E**). (**F**) displays final tumor volume, measured by caliper. (**G**) Number of days required for tumors to triple their volume, compared to baseline. (n=9-10). Error bars show Mean ± SEM. Statistical analysis was performed using one-way ANOVAs. **P<0.002, ***P<0.0002, and ****P<0.0001.

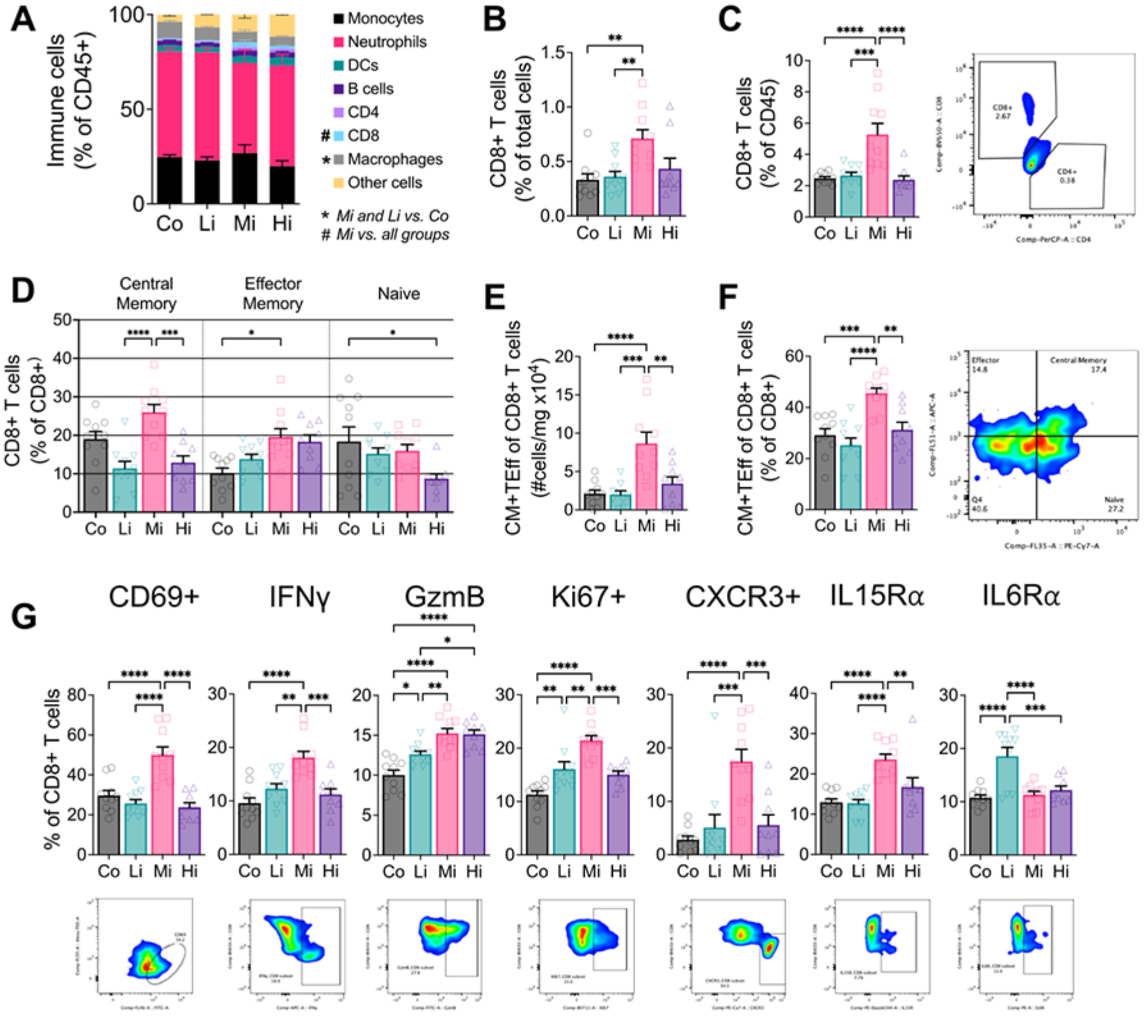

Figure S3. Immune cell profile and CD8+ T cell phenotype. (**A**) Average overall immune cell composition in the tumor microenvironment determined by flow cytometry (n = 7 to 10 per group) shows a significant reduction of Macrophages on Mi and Hi and an increase in CD8+ T cells only in Mi ExTr. CD8+ T cells are expressed as (**B**) a percentage of total cells, and (**C**) as a fraction of CD45+ cells, with representative gating (C, right panel). The developmental stage of CD8+ T cells expressed as (**D**) percentage of CD8+ T cells, displaying Central memory (CD62L+CD44+, left panel), Effector memory (CD62L-CD44+, medium panel) and Naïve (CD62L-CD44-, right panel). (**E-F**) Quantification of total effector cells (central + effector memory phenotypes), expressed in (E) absolute numbers or (F, left panel) as a fraction of CD8+ T cells, with representative gating (F, right panel). (**G**) Panel displaying the fraction of CD8+ T cells expressing activation marker CD69, cytotoxic function markers IFNγ and GzmB, proliferation marker Ki67, and markers involved in exercise-mediated tumor CD8+ T cell recruitment CXCR3, IL15Rα, and IL6Rα (respectively, left to right) (n=8-10) Representative gating for find in the bottom panel bellow the correspondent receptor (G, bottom panels). Error bars show Mean ± SEM. Statistical analysis was performed using one-way ANOVAs. *P<0.05, **P<0.002, ***P<0.0002, ****P<0.0001.

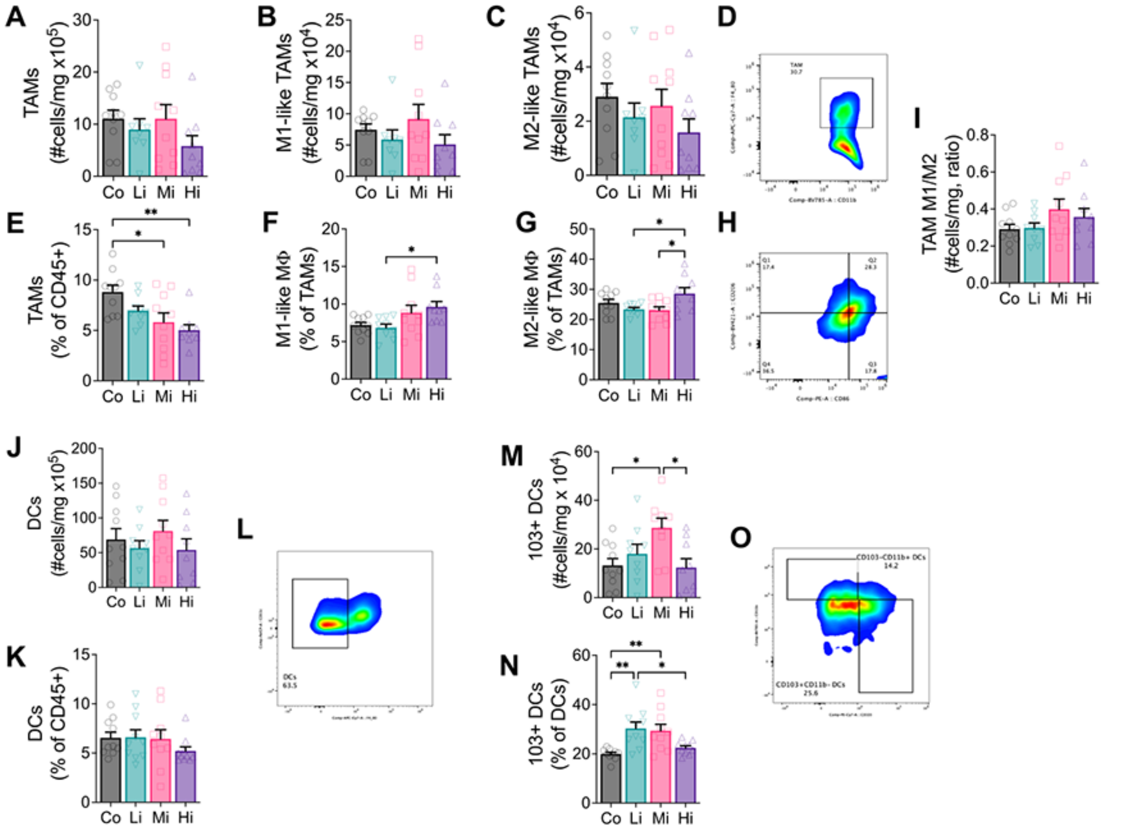

Figure S4. Role of exercise intensity macrophages and dendritic cells. (**A-D**) Absolute numbers of Tumor-associated Macrophages (TAM) (A), M1-like TAMs (B), and M2-like TAMs (C). Representative gating of macrophages (Mϕ) (D). (**E-I**) Relative numbers of TAMs (expressed as a function of CD45+ cells) (E), with a fraction of TAMs with M1-like (F) or M2-like (G) phenotype. Representative gating of macrophages (Mϕ) (H) and the ratio of M1 to M2 TAMs (I). (**J-L**) Dendritic cells are expressed as absolute number (J) and fraction of immune cells (K). Representative gating of dendritic cells (DCs) (L). (**M-O**) Absolute number (M) and fraction (N) of CD103+ DCs. Representative gating (O) (n=8-10). Error bars show Mean ± SEM. Statistical analysis was performed using one-way ANOVAs. *P<0.05, **P<0.002.

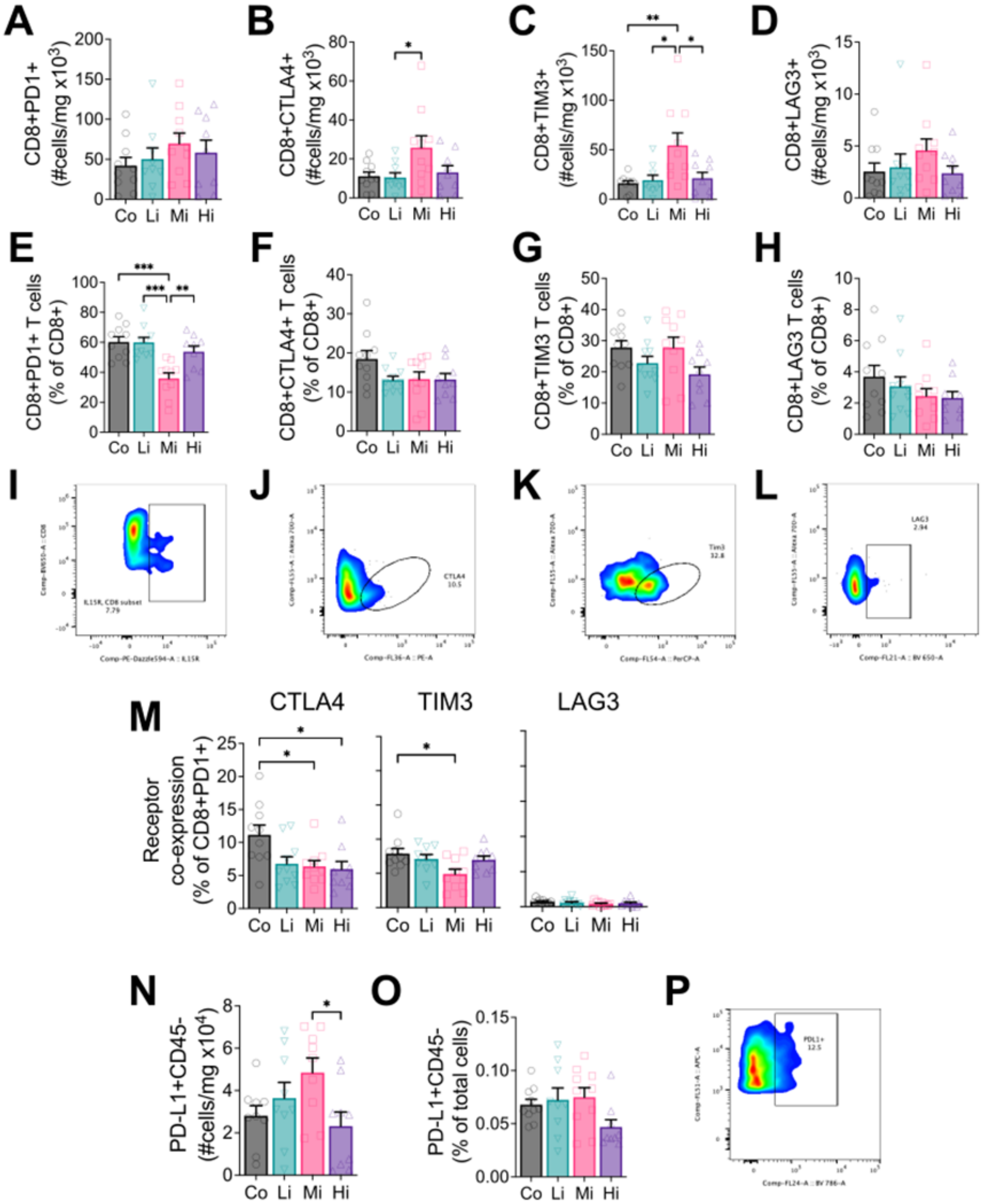

Figure S5. Role of exercise intensity on exhaustion markers in CD8+ T cells. (**A-D**) Absolute numbers of CD8+ T cells expressing PD1 (A), CTLA4 (B), TIM3 (C), and LAG3 (D). Fraction of CD8+ T cells expressing PD1 (E), CTLA4 (F), TIM3 (G), and LAG3 (H). (**I-L**) Representative gating for PD1 (I), CTLA4 (J), TIM3 (K), and LAG3 (L). (**M**) Fraction of CD8+ T cells co-expressing PD1 and CTLA4 (left panel), TIM3 (middle panel) and LAG 3 (right panel). (**N-P**) Absolute number (N) and fraction (O) of non-immune cells expressing PDL1, with representative gating (P) (n=8-10). Error bars show Mean ± SEM. Statistical analysis was performed using one-way ANOVAs. *P<0.05, **P<0.002.

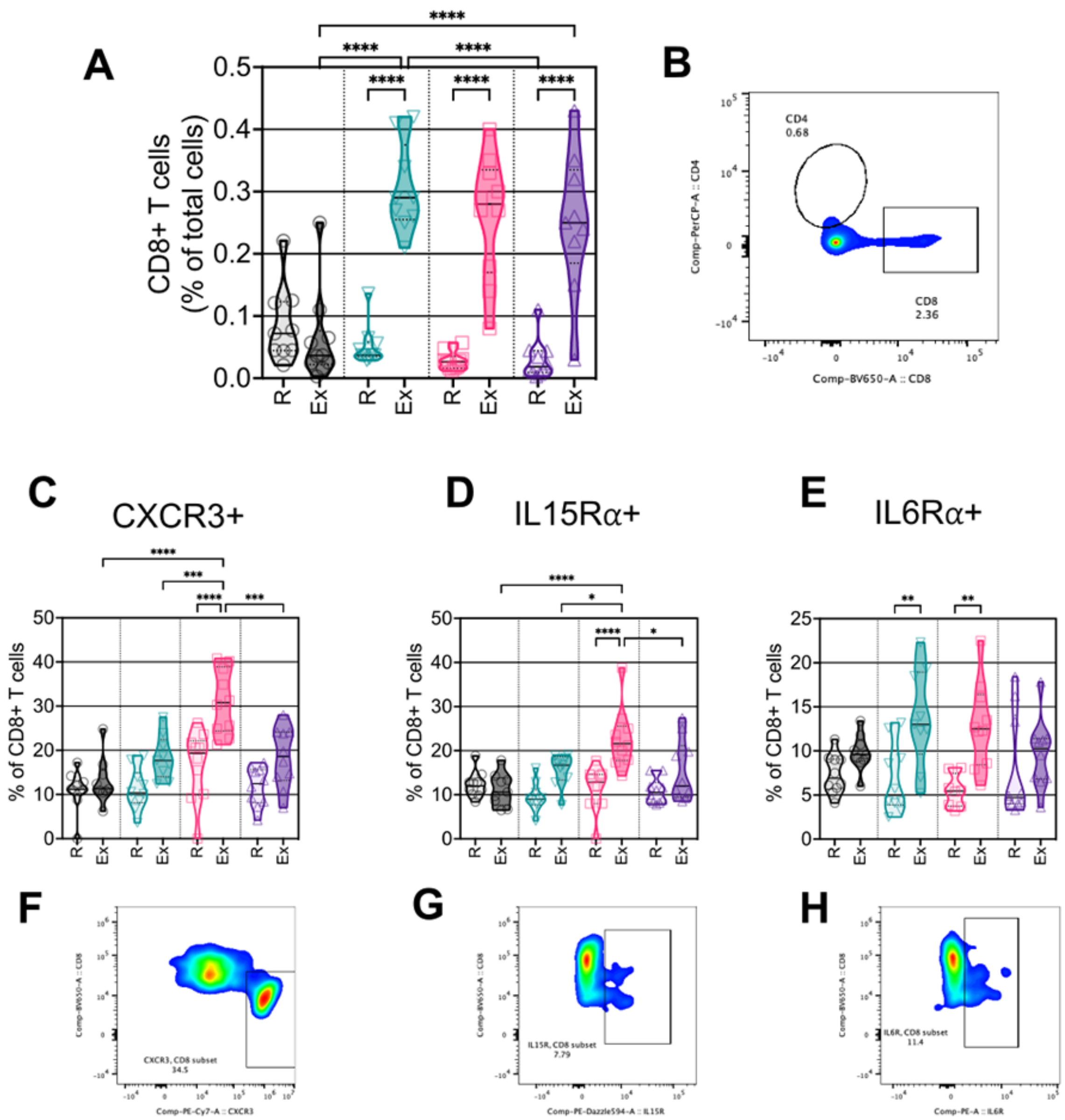

Figure S6. Relative expression exercise-mediated mobilization of CD8+ T cells to the circulation. (**A-B**) Relative number of CD8+ T cells in the circulation (A) and representative gating of CD8+ T cells in the circulation (B). (**C-H**) Among CD8+ T cells, expression (C) and representative gating (F) for CXCR3, expression (D) and representative gating (G) for IL15Rα, and expression (E) and representative gating (H) for IL6Rα. Light color represents the circulating levels at rest (R) and stronger color represents circulating levels of CD8+ T cells in response to exercise (Ex) (n=8-10). Error bars show Mean ± SEM. Statistical analysis was performed using one-way ANOVAs. *P<0.05, **P<0.002, ***P<0.0002, ****P<0.0001.
